# Timing of perioperative oral care and postoperative pneumonia: a propensity score–matched cohort study

**DOI:** 10.1186/s12903-026-08422-3

**Published:** 2026-04-25

**Authors:** Hiromi Nishi, Hideo Shigeishi, Susumu Horikoshi, Kiho Naganuma, Kouji Ohta, Yukio Yoshioka, Masaru Konishi, Saki Takesue, Minori Nakasato, Misako Tari, Mariko Maruyama, Sachiko Yamasaki, Nanako Ito, Kanako Yano, Miyuki Nakaoka, Noriyoshi Mizuno, Kotaro Tanimoto, Naoya Kakimoto, Hiroki Ohge, Hiroyuki Kawaguchi

**Affiliations:** 1 Department of General Dentistry, Hiroshima University Hospital, 1-2-3 Kasumi, Minami-ku, Hiroshima, 734-8553 Japan; 2 Department of Public Oral Health, Program of Oral Health Sciences, Graduate School of Biomedical and Health Sciences, Hiroshima University, Hiroshima, Japan; 3 Department of Clinical Practice and Support, Hiroshima University Hospital, Hiroshima, Japan; 4 Department of Oral and Maxillofacial Surgery, Kochi Medical School, Kochi University, Kochi, Japan; 5 Department of Oral and Maxillofacial Radiology, Hiroshima University Hospital, Hiroshima, Japan; 6 Department of Orthodontics and Craniofacial Developmental Biology, Graduate School of Biomedical and Health Sciences, Hiroshima University, Hiroshima, Japan; 7 Department of Periodontal Medicine, Graduate School of Biomedical and Health Sciences, Hiroshima University, Hiroshima, Japan; 8 Department of Advanced Prosthodontics, Graduate School of Biomedical and Health Sciences, Hiroshima University, Hiroshima, Japan; 9 Department of Oral Oncology, Graduate School of Biomedical and Health Sciences, Hiroshima University, Hiroshima, Japan; 10 Department of Oral and Maxillofacial Radiology, Graduate School of Biomedical and Health Sciences, Hiroshima University, Hiroshima, Japan; 11 Department of Infectious diseases, Hiroshima University Hospital, Hiroshima, Japan

**Keywords:** Postoperative pneumonia, Intervention timing, Perioperative oral care

## Abstract

**Background:**

Oral care is considered effective for preventing postoperative pneumonia (POP), though optimal timing for its implementation remains unclear. Opinions regarding the timing of oral care in clinical practice are conflicting, as some clinicians consider that reducing the oral bacterial load immediately before intubation is effective, whereas others are concerned that oral care performed just before surgery may promote bacterial dispersion and microaspiration. As a result, there is no consensus regarding the optimal timing of intervention. In addition, given the limited human resources and time available in perioperative care, identifying the timing that maximizes preventive effectiveness is important for optimizing clinical practice.

**Methods:**

This retrospective cohort study examined the associations of presence and timing of oral care with POP incidence following surgery under general anaesthesia. To account for potential confounding factors related to patient background and perioperative severity, propensity score matching and multivariate analyses were performed. POP, the primary outcome, was determined based on ICD-10 codes and initiation of antibiotic administration. Analysis was conducted using multivariate logistic regression.

**Results:**

Before propensity score matching, 1,783 patients were included in the analysis, from which 376 matched pairs were selected. Patients who underwent intervention had a lower POP incidence rate (1.6% vs. 4.3%), which was an independent risk-reducing factor (aOR 0.27, 95% CI 0.14–0.52). Furthermore, patients with intervention were divided into three groups (preoperative > 48-hour, preoperative ≤ 48-hour, postoperative), and univariate analysis showed that incidence rate was highest in the postoperative group (11.8%) and lowest in the preoperative ≤ 48 h group (2.0%). On the other hand, multivariate analysis showed that timing did not have a statistically significant effect, though ICU admission and tube feeding were identified as independent risk factors.

**Conclusion:**

To increase the effectiveness of intervention, optimization of planning, particularly timing, and accumulation of results for identifying high-risk patients are required. This study indicates that, when evaluating the effectiveness of perioperative oral care, it is necessary to consider structural factors related to disease severity rather than focusing solely on the timing of intervention, and that these factors should be incorporated into future considerations of perioperative oral care practice.

**Supplementary Information:**

The online version contains supplementary material available at 10.1186/s12903-026-08422-3.

## Introduction

Postoperative pneumonia (POP) is a major perioperative complication associated with prolonged hospital stay, increased mortality, and higher medical costs [1, 2]. Thus, POP prevention is considered important for improving the quality of perioperative care as well as optimizing healthcare resources, with several reports documenting its effectiveness presented. For example, among patients who underwent surgery for esophageal cancer, oral care provided during hospitalization was reported to reduce the risk of developing POP by approximately half [3]. Furthermore, analysis of patients who underwent cardiac valve surgery found a significant reduction in inflammatory markers such as CRP in patients who underwent oral care intervention, suggesting its contribution to control systemic inflammation [4].

Perioperative oral care includes professional mechanical tooth cleaning (PMTC), tongue cleaning, mouth rinsing, and moisturizing, with effectiveness determined by a combination of factors such as surgical procedure performed, type of patient, and timing. As for the procedure, it has been reported that oral care using chlorhexidine can reduce POP occurrence [5], while another study found that the rates of POP and surgical site infection (SSI) incidence did not increase even when PMTC was omitted during the COVID-19 pandemic period [6]. Additionally, there is growing evidence regarding the microbiological mechanisms involved. Periodontal pathogens, especially *P. gingivalis*, are frequently detected in saliva and tongue coating samples from patients with acute respiratory failure who require intensive care unit (ICU) admission, which indicates an association between specific bacterial species and severe respiratory impairment [7]. Furthermore, a higher postoperative oral bacterial count was found to be significantly associated with greater incidence of postoperative complications such as POP and SSI [8].

Although knowledge regarding related procedures, the oral environment, and microbiological factors has advanced, findings regarding intervention timing remain limited.

The oropharynx functions as an important reservoir of respiratory pathogens during the perioperative period, thus limiting the oral bacterial load immediately prior to intubation can result in a reduced number of bacteria that migrate to the lower respiratory tract [9]. Alternatively, performing oral care shortly before intubation may result in transient inoculation of the oral cavity and pharynx with bacteria and cleansing solutions, while microaspiration can also occur in some patients [10]. Therefore, oral care performed immediately prior to intubation has both advantages and disadvantages. In addition, other studies have shown an increased risk of POP during specific perioperative periods when aspiration and declines in airway defense mechanisms are more likely to occur [11, 12]. It is thus considered that the effectiveness of perioperative oral care can differ depending on implementation timing, which is a crucial factor to clarify in order to achieve optimal outcomes with limited human resources.

Furthermore, patients with a high oral bacterial count have been shown to have greater daily medical expenses [13], thus indicating that optimization of intervention timing is important from a health-economic perspective.

With this background information in mind, we speculated that the effectiveness of an intervention procedure can vary depending on its timing. The present study retrospectively examined findings of adult patients who underwent surgery under general anaesthesia at a single acute care hospital to reveal the relationship between presence or absence of perioperative oral care, and timing of its implementation (preoperative > 48-hour, preoperative ≤ 48-hour, postoperative) and occurrence of POP.

## Methods

### Study design

This was a retrospective observational study conducted at a single acute care hospital and the subjects enrolled were inpatients who underwent surgery under general anaesthesia between July 2021 and March 2022. It was conducted to evaluate the association between implementation of perioperative oral care and POP, and also examine the association between perioperative oral care timing and POP development.

### Study population

#### Inclusion criteria

Patients who were 20 years old or older and underwent surgery under general anaesthesia at our hospital, and whose hospital stay was longer than one week were considered eligible for enrolment.

#### Exclusion criteria

**Fig. 1:**
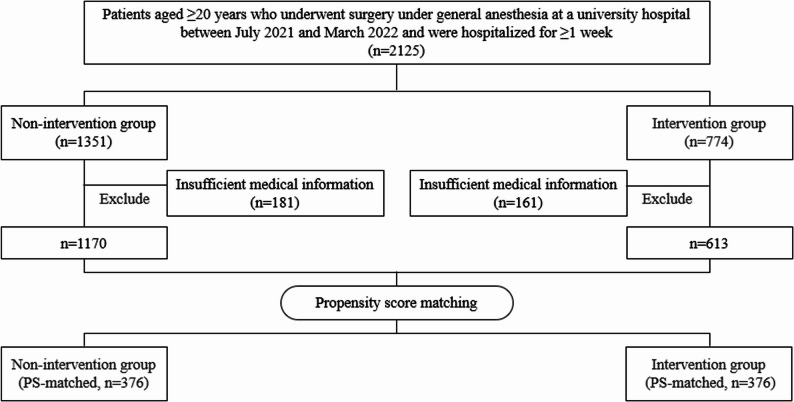
Study flow diagram. Patient screening, eligibility assessment, and reasons for exclusion

Patients under 20 years old, discharged within one week, with a current diagnosis of pneumonia at the time of admission, receiving artificial ventilation due to a tracheotomy, with incomplete medical records, or admitted to the emergency department prior to surgery were excluded. After applying the inclusion and exclusion criteria, a total of 1,783 subjects were included in the final analysis (Fig. 1).

### Data sources

For this study, the following information was extracted from the Diagnosis Procedure Combination (DPC) database and electronic medical records (EMR) : surgical information including anaesthesia method, surgical procedure, operative time, and blood loss; ward management information including use of mechanical ventilation, tube feeding, oral intake status (regular diet/soft or pureed diet), and oral care intervention; and medication information including type of intravenous antibiotic and administration history. Due to the large number of cases in the database, medical records and radiological examination records were not individually reviewed. Rather, uniform assessments based on the obtained information were performed using the same criteria.

### Outcome definitions

The primary outcome for analysis was onset of POP. The International Classification of Diseases, 10th revision (ICD-10), was used for disease identification, with the target pneumonia codes J12-J18 and J69. Date of surgery was the time reference point. When multiple operations were performed for an individual patient during the same hospitalization, the first surgery within the study period was considered as the starting point for analysis.

Patients already assigned a pneumonia ICD-10 code at the time of admission were excluded. Subsequently, POP was defined as cases satisfying both of the following conditions simultaneously: a new ICD-10 code for pneumonia added within one week of the surgery date and intravenous antibiotic administration initiated on the same day the ICD-10 code was added. The criterion of onset within one week from the date of surgery conforms to the European Perioperative Clinical Outcome definitions provided by the European Society of Anesthesiology and European Society of Intensive Care Medicine [14, 15]. Inclusion based on ICD code alone may incorporate cases in which pneumonia was suspected but not ultimately diagnosed, thus diagnosis specificity was improved by including initiation of antibiotic administration. Indeed, it has been reported that use of an algorithm to extract pneumonia using ICD codes alone often exhibits sensitivity below 80%, while a combination of indicators such as antimicrobial administration improves diagnostic accuracy [16]. Other studies have noted that when infection cases were extracted from large-scale data, diagnostic accuracy was low when disease codes alone were used, whereas incorporation of additional of information, such as antimicrobial administration or laboratory test results, enhanced precision [16, 17]. Intravenous antibiotics considered included ampicillin-sulbactam, piperacillin-tazobactam, cefazolin, ceftriaxone, cefotaxime, ceftazidime, cefepime, meropenem, imipenem/cilastatin, azithromycin, levofloxacin, clindamycin, and metronidazole, chosen in accordance with the American Thoracic Society/Infectious Diseases Society of America (ATS/IDSA) clinical practice guidelines for community-acquired pneumonia and for hospital-acquired and ventilator-associated pneumonia [18, 19].

### DPC database

The DPC database contains standardized medical and billing information widely used in acute care hospitals in Japan, including patient information, comorbidities, medical procedures, surgical procedures and interventions, outcomes, medical resources such as medications, tests, imaging, and healthcare costs, with ICD-10 employed for disease classification [20]. A previous study of DPC data for proximal femoral fracture cases noted that early operations and introduction of regional clinical pathways were associated with shorter hospital stays [21]. Thus, the data are widely used for research performed to evaluate disease-specific outcomes and appropriate use of medical resources.

### Exposure definition

For the present study, perioperative oral care was defined as overall oral management performed by dental staff upon request during the perioperative period. Standard procedures used included cleaning of tooth surfaces and dentures, wiping of oral mucosa and tongue coating, mouth rinsing or moisturizing, periodontal and caries treatment including tooth extraction as needed, and self-care instruction. Based on timing of perioperative oral care implementation, the analyzed patients were divided into three groups; preoperative > 48-hour (final oral care performed more than 48 h before surgery, with intervention occurring 2–7 days prior to surgery), preoperative ≤ 48-hour (final oral care performed within 48 h of surgery, i.e., day before or day of surgery), and postoperative group (initial oral care performed following surgery). Patients with multiple interventions performed were assigned to a group based on the timing of the final oral care. Postoperative oral care was performed for all patients on the day after surgery and third postoperative day, with the same timing and content of care provided. The present study examined the timing of preoperative rather than postoperative intervention as an exposure factor.

### Statistical analysis

#### Descriptive statistics

All statistical analyses were conducted using a two-sided test with a significance level of α = 0.05, using JMP^®^ Pro 17 (JMP Statistical Discovery LLC, Cary, NC, USA). Continuous variables are presented as mean ± standard deviation or median values. Intergroup comparisons were performed using Student’s t-test or the Mann-Whitney U test, depending on data distribution. Categorical variables are presented as number of patients (%), and between-group comparisons were performed using a chi-square test or Fisher’s exact test. Estimates were calculated by logistic regression and presented with 95% CI values.

#### Analysis 1: association between perioperative oral care and postoperative pneumonia

First, the patients were divided into those with (*n* = 376) and without (*n* = 376) perioperative oral care. To adjust for confounding factors, 1:1 nearest neighbour propensity score matching was performed. The propensity score model comprised predefined major background factors, including age, sex, BMI category, Charlson Comorbidity Index (CCI), operative time, blood loss, ICU admission, use of mechanical ventilation, tube feeding, and oral intake status (regular diet/soft or pureed diet). The calliper was set to 0.2 times the standard deviation of the logit-transformed propensity score and no replacement was performed. Covariate balance was assessed based on standardized mean difference (SMD), with values ≤ 0.1 considered to indicate a good balance. In addition, receiver operating characteristic (ROC) analysis of the propensity score model was performed and the c-statistic (area under the curve; AUC) was calculated. These results are shown in Table 1.

**Table 1:** Baseline characteristics after propensity score matching

Parameter	Non-intervention group n=376	Intervention group n=376	SMD*
Age, years, mean ± SD	64.4±16.4	65.0±15.7	0.037
Gender, male, n (%)	200 (53.2)	216 (57.5)	0.086
BMI			
<18.5, n (%)	35 (9.3)	36 (9.6)	0.010
18.5-24.9, n (%)	231 (61.4)	225 (59.8)	0.033
25-29.9, n (%)	82 (21.7)	88 (23.4)	0.041
≥30, n (%)	28 (7.5)	27 (7.2)	0.011
CCI, mean ± SD	0.9±1.4	1.0±1.5	0.069
Operative time ≥278 min, n (%)	80 (21.3)	81 (21.5)	0.005
Blood loss ≥150 mL, n (%)	107 (28.5)	115 (30.6)	0.046
ICU admission, n (%)	53 (14.1)	48 (12.8)	0.038
Use of mechanical ventilation, n (%)	87 (23.1)	84 (22.3)	0.019
Tube feeding, n (%)	16 (4.3)	16 (4.3)	0.000
Oral intake status (regular diet/soft or pureed diet), n (%)	5 (1.3)	8 (2.1)	0.062

*SMD standardized mean difference

Univariate logistic regression of the matched data was initially performed, followed by multivariate logistic regression analysis using the predefined major background factors to determine the adjusted odds ratio (aOR). Previous large-scale studies have reported indicators reflecting perioperative systemic status and surgical invasiveness as independent risk factors for aspiration pneumonia [22, 23], which were thus used for estimating propensity score and selecting covariates for multivariate analysis in the present investigation. Tube feeding and impaired swallowing function have been shown to be major risk factors for aspiration pneumonia [24, 25]. In addition, other factors such as ICU admission, use of mechanical ventilation, and tube feeding are noted in ATS/IDSA guidelines as criteria for classifying severity and management of hospital-acquired pneumonia and ventilator-associated pneumonia [18], thus those were used as adjustment variables. Covariates used for propensity score estimation and multivariate analysis were age, sex, BMI category, CCI, operative time, blood loss, ICU admission, use of mechanical ventilation, tube feeding, and oral intake status (regular diet/soft or pureed diet). Operative time and blood loss were dichotomized according to cutoff values determined by ROC curve analysis, set at 278 min and 150 mL, respectively. The goodness-of-fit of the multivariable logistic model was evaluated using a lack-of-fit test.

#### Analysis 2: association of perioperative oral care timing and POP

Next, the association of oral care timing and occurrence of POP in patients who underwent perioperative oral care was evaluated. Because oral variables were also included as candidate factors, 49 patients with insufficient oral information were excluded from this analysis. The patients were divided into the preoperative > 48-hour, preoperative ≤ 48-hour, and postoperative groups. Oral variables assessed were number of missing teeth ≥ 6, coated tongue, and oral dryness, with selection limited to variables with relatively low rates of missing data. Coated tongue was evaluated using the modified Oral Assessment Guide (mOAG), with scores of 2 and 3 indicating the presence of tongue coating [26]. Oral dryness was assessed using an oral moisture-checking device (Mucus^®^), with values < 27.0 indicating dryness [27]. Using the preoperative ≤ 48-hour group as the reference, univariate logistic regression was used to determine odds ratios for each group. Subsequently, multivariate logistic regression was performed to calculate aOR, with the predefined key background factors entered as covariates. These covariates included age, sex, BMI category, CCI, operative time, blood loss, ICU admission, use of mechanical ventilation, tube feeding, oral intake status (regular diet/soft or pureed diet), number of missing teeth ≥ 6, coated tongue, and oral dryness. For this analysis as well, the goodness-of-fit of the multivariable logistic model was evaluated using a lack-of-fit test.

## Results

### Study population and baseline characteristics

A total of 2,125 patients who underwent surgery under general anaesthesia during the study period were screened. The patient selection process, including inclusion and exclusion criteria, is presented in Fig. 1. The departments involved, number of patients, and incidence of postoperative pneumonia in the overall analytic cohort before propensity score matching are shown in Supplementary Table S1.

#### Analysis 1: association between perioperative oral care and postoperative pneumonia

Propensity score matching used to determine the relationship between presence or absence of perioperative oral care and POP resulted in a total of 752 patients, of whom 376 underwent intervention and 376 did not. Predefined major background factors were well-balanced between the groups, including age, sex, BMI category, CCI, operative time, blood loss, ICU admission, use of mechanical ventilation, tube feeding, and oral intake status (regular diet/soft or pureed diet), with those variables showing SMD ≤ 0.1. These baseline characteristics are summarized in Table 1. Baseline characteristics before propensity score matching performed in Analysis 1, including the number and proportion of POP cases in both groups, are shown in Supplementary Table S2. The c-statistic (AUC) of the propensity score model was 0.755 and the ROC curve is presented in Supplementary Figure S1.

**Fig. 2:**
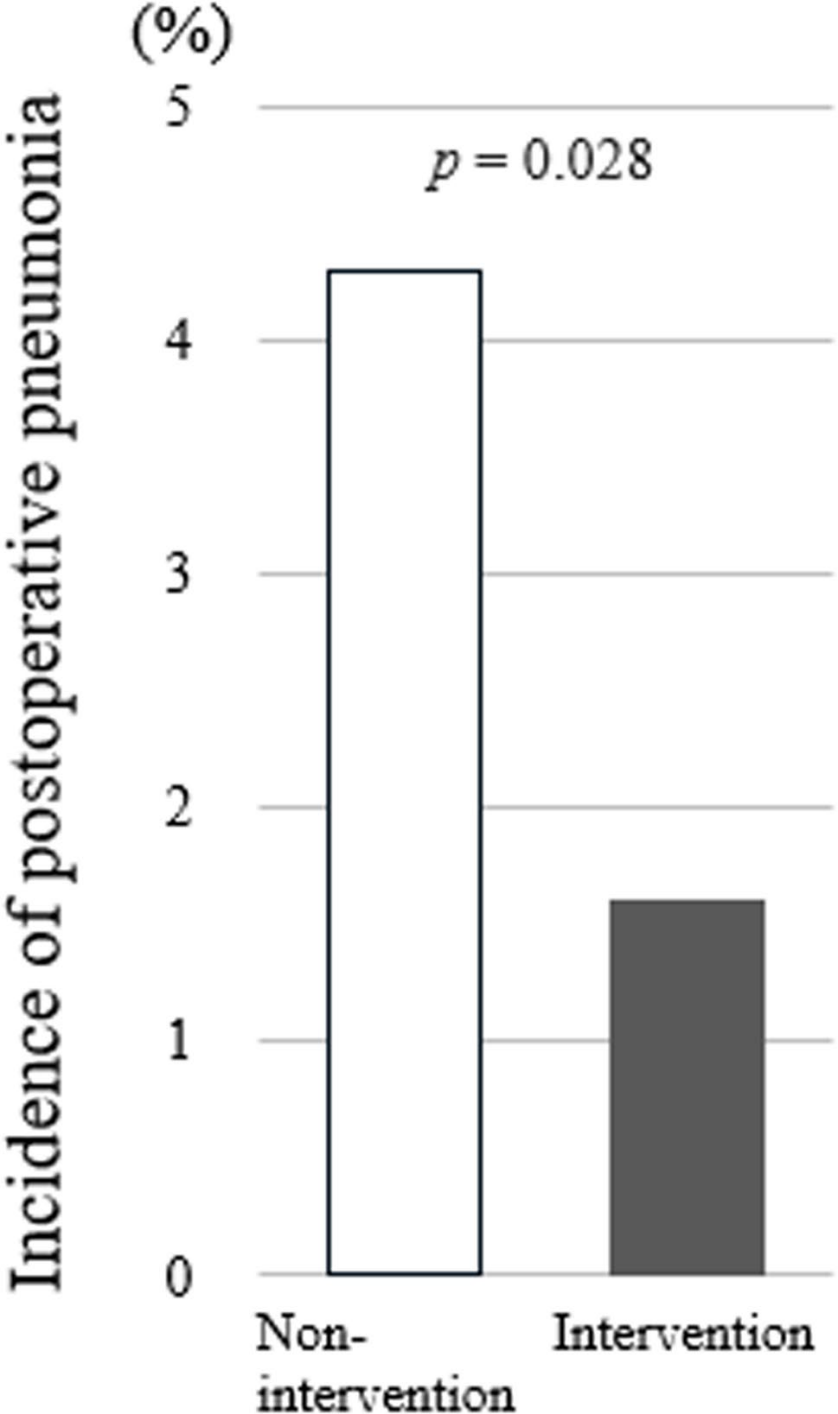
Incidence of postoperative pneumonia associated with perioperative oral care. In the propensity score–matched cohort (no intervention, *n* = 376; intervention, *n* = 376), postoperative pneumonia was significantly less frequent in patients who underwent intervention (chi-square test, *p* = 0.028)

In the propensity score-matched cohort, the incidence of POP was significantly lower in patients who underwent intervention (1.6%) as compared to those who did not (4.3%) (*p* = 0.028). These results are presented in Fig. 2. Multivariate logistic regression analysis showed that perioperative oral care was independently associated with a reduced risk of POP (aOR 0.27, 95% CI 0.14–0.52, *p* < 0.001). Furthermore, operative time ≥ 278 min, blood loss ≥ 150 mL, ICU admission, use of mechanical ventilation, and tube feeding were factors significantly associated with POP onset. No significant lack of fit was observed in the multivariable model (*p* = 1.000). These results are shown in Table 2.

**Table 2:** Results of univariable and multivariable logistic regression analyses to identify risk factors associated with postoperative pneumonia

	Univariable analysis			Multivariable analysis		
	OR	95% CI	*P*-value	aOR	95% CI	*P*-value
Age	1.03	1.00-1.07	0.073	1.02	1.00-1.03	0.080
Gender, male	2.82	1.10–8.65	0.030*	1.48	0.86–2.58	0.159
BMI			0.111			0.914
<18.5	0.45	0.03–2.20	0.437	0.77	0.30–1.96	0.578
18.5–24.9	1 (Ref)	-		1 (Ref)		
25-29.9	2.97	1.23–7.01	0.013*	1.17	0.64–2.17	0.608
≥30	0.60	0.03–2.94	0.617	1.05	0.38–2.90	0.931
CCI	1.37	1.12–1.63	0.001*	1.08	0.89–1.29	0.423
Operative time ≥ 278 min	4.68	1.98–11.29	< 0.001*	3.53	1.87–6.65	< 0.001*
Blood loss ≥ 150 mL	2.97	1.26–7.14	0.013*	2.14	1.16–3.95	0.015*
ICU admission	4.80	1.93–11.45	0.001*	2.65	1.17–5.98	0.019*
Use of mechanical ventilation	4.31	1.83–10.39	0.001*	4.66	2.18–9.97	< 0.001*
Tube feeding	10.15	3.42–27.01	< 0.0001*	3.52	1.66–7.46	0.001*
Oral intake status (regular diet/soft or pureed diet)	2.85	0.15–15.53	0.389	1.48	0.34–6.34	0.601
Perioperative oral care	0.36	0.13–0.90	0.028*	0.27	0.14–0.52	< 0.001*

*OR* odds ratio, *aOR* adjusted odds ratio, *CI* confidence interval

*P < 0.05

#### Analysis 2: association between timing of perioperative oral care and postoperative pneumonia

**Fig. 3:**
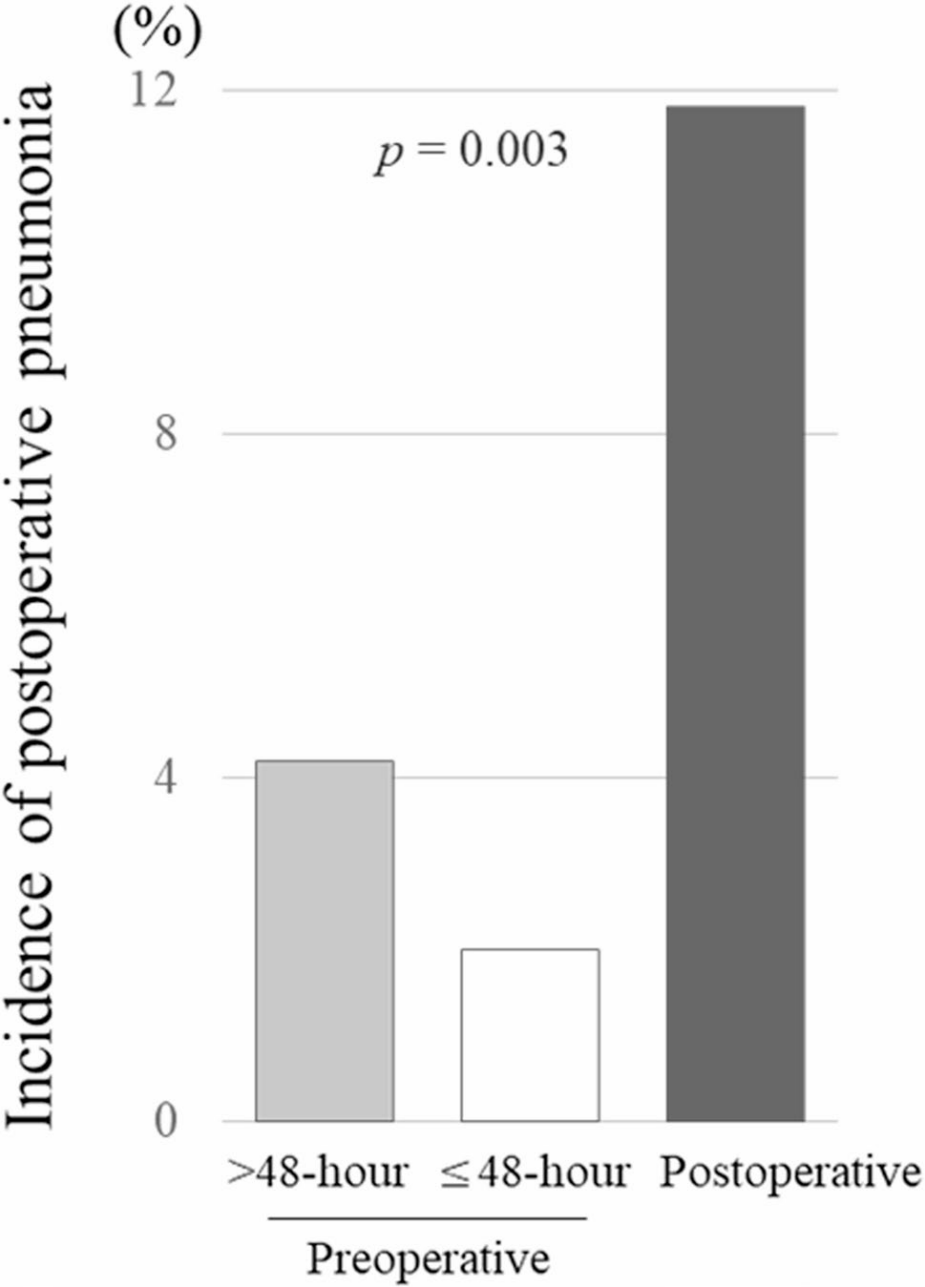
Incidence of postoperative pneumonia in the three groups. The incidence of postoperative pneumonia was significantly different among the groups (chi-square test, *p* = 0.003)

Of the 613 patients who underwent perioperative oral care in Analysis 1, 49 with insufficient oral information were excluded, thus 564 patients were categorized into three groups according to timing of the oral care procedure; preoperative > 48-hour (*n* = 190), preoperative ≤ 48-hour (*n* = 247), and postoperative (*n* = 127). Notably, nearly all patients who underwent preoperative oral care also received postoperative oral care as part of the standard postoperative oral care protocol. Among the baseline characteristics examined(summarized in Supplementary Table S3), ICU admission, mechanical ventilation usage, tube feeding, and oral dryness showed significant differences among the three groups, while the incidence of POP also showed a significant difference among them (*p* = 0.003). These results are presented in Fig. 3.

Univariate analysis indicated that the postoperative group had a significantly higher risk of POP development as compared with the preoperative ≤ 48-hour group (OR 6.48, 95% CI 2.44–20.33, *p* = 0.0001). There was also an increase in risk in the preoperative > 48-hour group as compared with the preoperative ≤ 48-hour group, though it was not significant (OR 2.13, 95% CI 0.70–7.14, *p* = 0.184). Multivariate logistic regression analysis showed that the postoperative group had a higher risk of POP development than the preoperative ≤ 48-hour group, though the difference was not statistically significant (aOR 3.26, 95% CI 0.85–7.41, *p* = 0.059). In contrast, postoperative ICU admission (aOR 5.68, 95% CI 1.73–18.64, *p* = 0.004) and tube feeding (aOR 7.32, 95% CI 2.20–24.27, *p* = 0.001) were significantly associated with development of POP. There was no significant lack of fit observed in the multivariable model (*p* = 1.000). These results are shown in Table 3.

**Table 3:** Results of univariable and multivariable logistic regression analyses for postoperative pneumonia according to timing of perioperative oral care

	Univariable analysis			Multivariable analysis		
	OR	95% CI	*P*-value	aOR	95% CI	*P*-value
Age	1.03	1.00-1.06	0.082	1.02	0.99–1.06	0.301
Gender, male	1.22	0.55–2.62	0.611	2.25	0.79–6.40	0.128
BMI						
<18.5	1.52	0.43–4.11	0.457	0.99	0.27–3.65	0.99
18.5–24.9	1 (Ref)			1 (Ref)		
25-29.9	3.80	1.11–23.79	0.031༊	0.11	0.01–0.97	0.047
≥30	0.95	0.15–3.35	0.948	0.4	0.06–2.52	0.331
CCI	1.13	0.89–1.38	0.300	1.2	0.87–1.58	0.253
Operative time ≥ 278 min	1.35	0.57–3.72	0.517	1.59	0.49–5.15	0.440
Blood loss ≥ 150 mL	1.72	0.74–4.44	0.212	2.43	0.81–7.32	0.115
ICU admission	2.51	1.06–6.92	0.035*	5.68	1.73–18.64	0.004*
Use of mechanical ventilation	2.15	0.97–5.27	0.073	3.3	0.90–12.08	0.072
Tube feeding	8.07	3.69–18.09	< 0.0001*	7.32	2.20-24.27	0.001*
Oral intake status (regular diet/soft or pureed diet)	1.72E-06	0-6.11	0.395	not estimable		0.998
Number of missing teeth ≥ 6	1.05	0.46–2.52	0.911	0.88	0.31–2.45	0.801
Oral dryness	1.65	0.73–3.83	0.230	1.20	0.43–3.33	0.727
Coated tongue	2.26	0.73–5.89	0.227	3.06	0.87–10.80	0.082
Timing of perioperative oral care						
Preoperative > 48-hour	2.13	0.70–7.14	0.184	2.4	0.61–9.38	0.209
Preoperative ≤ 48-hour	1 (Ref)			1 (Ref)		
Postoperative	6.48	2.44–20.33	0.0001*	3.26	0.85–7.41	0.059

*OR* odds ratio, *aOR* adjusted odds ratio, *CI* confidence interval

*P < 0.05

## Discussion

The objective of this study was to clarify the optimal timing of perioperative oral care for prevention of POP. During the perioperative period, the oropharynx functions as a significant reservoir of respiratory pathogens, and intervention timing has both advantages and disadvantages. Furthermore, in order to maximize efficiency in clinical settings with limited human resources, it is important to determine which patients should receive intervention and its optimal timing. With these factors in mind, the present retrospective observational study was conducted to verify the effectiveness of perioperative oral care as well as impact associated with timing. Patients who underwent surgery under general anaesthesia in an acute care hospital were analyzed. It was found that the incidence of POP was significantly lower in patients with as compared to without intervention (aOR 0.27, 95% CI 0.14–0.52), demonstrating that perioperative oral care contributes to prevention of POP. These findings are consistent with those noted in previous studies [22, 28, 29] and reaffirm the efficacy of perioperative oral care. On the other hand, while previous investigations have suggested that perioperative oral care is effective in preventing POP, clear evidence regarding the timing of its implementation remains lacking [30]. To focus on this issue, we analyzed the effect of perioperative oral care timing on incidence rate of POP.

Univariate analysis results showed that patients who received oral care postoperatively had a significantly higher risk of developing POP as compared with those who underwent preoperative oral care. On the other hand, there was no significant difference between the preoperative > 48-hour and preoperative ≤ 48-hour groups, indicating that implementation of oral care preoperatively was effective regardless of its timing. However, the preoperative ≤ 48-hour group showed a tendency for a lower incidence of POP as compared to the preoperative > 48-hour group. Furthermore, multivariate analysis showed that ICU admission and use of tube feeding, factors related to overall condition, had a stronger influence on risk of developing POP than timing of oral care. These findings indicate that the association identified by univariate analysis was confounded by disease severity.

In clinical settings, there is often not enough time to provide oral care for patients with severe conditions prior to surgery. As a result, initial intervention is often delayed until after surgical procedures, which increases the likeliness of POP occurrence. This may explain why oral care timing was not found to be significant in multivariate analysis. Because delays in intervention are inherently linked to patient condition severity, making it difficult to completely eliminate the impact, careful attention must be given to disease severity when assessing the true effect of oral care timing. Additional studies are thus needed to develop new procedures that are applicable even in severe cases, as well as for designing interventions tailored to disease severity and evaluation of optimal timing.

In observational studies, residual confounding is often unavoidable and other approaches should be considered to clarify causal relationships. In recent years, target trial emulation (TTE) as a countermeasure against unmeasured confounding has gained attention [31, 32]. TTE is a method used to analyze observational data by first clearly defining its components, such as intervention, comparison, and outcome, as well as others, as if conducting a randomised controlled trial. For the present study, propensity score matching and multivariate analysis were used to reduce the effects of confounding. However, future prospective studies that incorporate TTE are needed to evaluate the effects of perioperative oral care in greater detail.

The present study also found that patients expected to be admitted to an ICU or receive tube feeding carry an increased risk of POP development. For such high-risk cases, it is important to develop effective techniques aimed at reducing the burden of oral biofilm from the preoperative to postoperative period and increase the frequency of care. In recent years, use of EMR for automated surveillance of non-ventilator-associated hospital-acquired pneumonia and early identification of high-risk patients for facilitation of preventive interventions has been tested in multiple centres [33]. In order to develop procedures that maximize preventive effectiveness with minimal human resources, it will be necessary to accumulate evidence related to optimising the timing and frequency of pre- and postoperative procedures, as well as clinical and microbiological indicators for accurate identification of high-risk patients. In recent years, models that use EMR to predict pulmonary complications after surgery including femoral fracture surgery in elderly patients have been presented [34, 35]. It is anticipated that similar methodologies could be employed to construct risk prediction models for infection prevention with use of perioperative oral care and thereby enhance the efficiency of preventive intervention methods.

This study has several limitations. First, it was conducted in a retrospective manner at a single facility and the clinical practice methods used were generally standardized; however, it was not possible to completely eliminate possible confounding factors. Furthermore, pneumonia cases were defined using both diagnosis of pneumonia and antimicrobial therapy initiation, though it may not have been possible to adequately distinguish cases of mild pneumonia or those that received treatment for other infections. In addition, the number of events was limited as compared with number of variables in the multivariable models, which should be considered when interpreting the results. Finally, frequency and quality of oral care performed by the patients themselves were not verified, and differences among them may have influenced the results.

Although univariate analyses suggested an association between oral care timing and POP incidence, this association was attenuated after adjustment, indicating substantial confounding by disease severity and feasibility of preoperative intervention.

Furthermore, the timing of such care does not need to be strictly limited to immediately prior to surgery to achieve a preventive effect. Accordingly, in clinical settings it may be practical to provide at least one session of preoperative oral care, with the timing determined based on patient condition and available human resources. For patients at high risk of developing pneumonia, in addition to preoperative measures it is important to consider the frequency and content of postoperative care. Because severe cases are more likely to experience delayed initiation of oral care until after surgery, severity-tailored perioperative protocols should be developed and evaluated in future studies. To add to these prospective research findings, advanced studies focused on related oral microorganisms will be necessary to identify more effective intervention methods.

## Conclusions

The present findings indicate that preoperative oral care is effective for preventing POP. Although univariate analyses suggested an association between oral care timing and POP incidence, that association was attenuated after adjustment, indicating substantial confounding by disease severity and feasibility of preoperative intervention. These results suggest that the preventive effect of perioperative oral care does not depend solely on its timing and that patient condition should be considered when implementing intervention in clinical settings.

## Supplementary Information

Supplementary Material 1.

Supplementary Material 2. Figure S1. Receiver operating characteristic curve of propensity score model in Analysis 1. The c-statistic (AUC) was 0.755

## Data Availability

The datasets used and/or analyzed during the current study are available from the first author upon reasonable request.

## Abbreviations

ATS/IDSA: American thoracic society/Infectious diseases society of america
CCI: Charlson Comorbidity Index
DPC: Diagnosis procedure combination
EMR: Electronic medical records
ICU: Intensive care unit
PMTC: Professional mechanical tooth cleaning
POP: Postoperative pneumonia
SMD: Standardized mean difference
SSI: Surgical site infection
TTE: Target trial emulation
