# Supplementary material for: Timing of perioperative oral care and postoperative pneumonia: a propensity score–matched cohort study
Source: BMC Oral Health. 2026 Apr 25;26:1092. doi: 10.1186/s12903-026-08422-3 (PMC13285105; doi:10.1186/s12903-026-08422-3)
Supplement: Supplementary file 1 — Supplementary Material 1. [file 12903_2026_8422_MOESM1_ESM.docx]

**Supplementary Table S1. Department distribution and incidence of POP in overall analytic cohort before propensity score matching**

| Department | Patients, n | POP, n | POP incidence, % |
| --- | --- | --- | --- |
| Gastrointestinal surgery (excluding esophageal surgery) | 446 | 15 | 3.4 |
| Orthopaedic surgery | 347 | 11 | 3.2 |
| Cardiovascular surgery | 204 | 13 | 6.4 |
| Head and neck/esophageal surgery | 146 | 20 | 13.7 |
| Urology | 135 | 2 | 1.5 |
| Breast surgery | 135 | 0 | 0.0 |
| Neurosurgery | 129 | 8 | 6.2 |
| Thoracic/respiratory surgery | 98 | 9 | 9.2 |
| Gynecology | 96 | 2 | 2.1 |
| Dermatology | 40 | 2 | 5.0 |
| Other departments | 7 | 0 | 0.0 |

**Supplementary Table S2. Baseline characteristics of two groups before propensity score matching in Analysis 1**

| Parameter | Non-intervention group | Intervention group | P-value |
| --- | --- | --- | --- |
|  | n=1,170 | n=613 |  |
| Age, years, mean ± SD | 59.6±17.0 | 67.7±13.9 | <0.0001^＊^ |
| Gender, male, n (%) | 551 (47.1) | 383 (62.5) | <0.0001^＊^ |
| BMI |  |  | 0.0003^＊^ |
| <18.5, n (%) | 90 (7.7) | 66 (10.8) |  |
| 18.5-24.9, n (%) | 677 (57.9) | 381 (62.2) |  |
| 25-29.9, n (%) | 308 (26.3) | 127 (20.7) |  |
| ≥30, n (%) | 95 (8.1) | 39 (6.3) |  |
| CCI, mean ± SD | 0.6±1.2 | 1.2±1.5 | <0.0001^＊^ |
| Operative time ≥278 min, n (%) | 360 (30.8) | 319 (52.1) | <0.0001^＊^ |
| Blood loss ≥150 mL, n (%) | 454 (38.8) | 376 (61.3) | <0.0001^＊^ |
| ICU admission, n (%) | 89 (7.6) | 173 (28.2) | <0.0001^＊^ |
| Use of mechanical ventilation, n (%) | 145 (12.4) | 241 (39.3) | <0.0001^＊^ |
| Tube feeding, n (%) | 40 (3.4) | 86 (14.1) | <0.0001^＊^ |
| Oral intake status (regular diet/soft or pureed diet), n (%) | 26 (2.2) | 11 (1.8) | 0.519 |
| POP, n (%) | 54 (4.6) | 28 (4.6) | 0.964 |

*P <0.05

**Supplementary Table S3. Baseline characteristics of three timing groups**

| Parameter | Preoperative > 48-hour  n=190 | Preoperative 　 ≤ 48-hour  n=247 | Postoperative  n=127 | P-value |
| --- | --- | --- | --- | --- |
| Age, years, mean ± SD | 70.5±14.5 | 68.0±14.1 | 69.2±15.1 | 0.186 |
| Gender, male, n (%) | 118 (62.1) | 143 (57.9) | 87 (68.5) | 0.131 |
| BMI |  |  |  | 0.563 |
| <18.5, n (%) | 21 (11.1) | 20 (8.1) | 16 (12.7) |  |
| 18.5-24.9, n (%) | 115 (60.5) | 156 (63.2) | 70 (55.6) |  |
| 25-29.9, n (%) | 37 (19.5) | 56 (22.7) | 30 (23.8) |  |
| ≥30, n (%) | 17 (9.0) | 15 (6.1) | 10 (7.9) |  |
| CCI, mean ± SD | 1.5±1.4 | 1.1±1.5 | 1.1±1.4 | 0.177 |
| Operative time ≥278 min, n (%) | 38 (20.5) | 70 (29.2) | 40 (32.5) | 0.051 |
| Blood loss ≥150 mL, n (%) | 53 (30.6) | 89 (38.9) | 52 (44.4) | 0.057 |
| ICU admission, n (%) | 100 (52.6) | 84 (34.0) | 40 (31.5) | <0.001* |
| Use of mechanical ventilation, n (%) | 125 (65.8) | 106 (42.9) | 77 (60.6) | <0.001* |
| Tube feeding, n (%) | 18 (9.5) | 28 (11.3) | 46 (36.2) | <0.001* |
| Oral intake status (regular diet / soft or pureed diet), n (%) | 3 (1.6) | 1 (0.4) | 3 (2.4) | 0.223 |
| Number of missing teeth ≥6, n (%) | 119 (62.6) | 152 (61.5) | 84 (66.1) | 0.677 |
| Oral dryness, n (%) | 61 (33.2) | 90 (38.3) | 78 (63.4) | <0.001* |
| Coated tongue, n (%) | 22 (12.0) | 23 (9.8) | 14 (11.4) | 0.762 |
| POP, n (%) | 8 (4.2) | 5 (2.0) | 15 (11.8) | 0.003* |

*P <0.05
