# Supplementary figures and images for: Timing of perioperative oral care and postoperative pneumonia: a propensity score–matched cohort study
Source: BMC Oral Health. 2026 Apr 25;26:1092. doi: 10.1186/s12903-026-08422-3 (PMC13285105; doi:10.1186/s12903-026-08422-3)

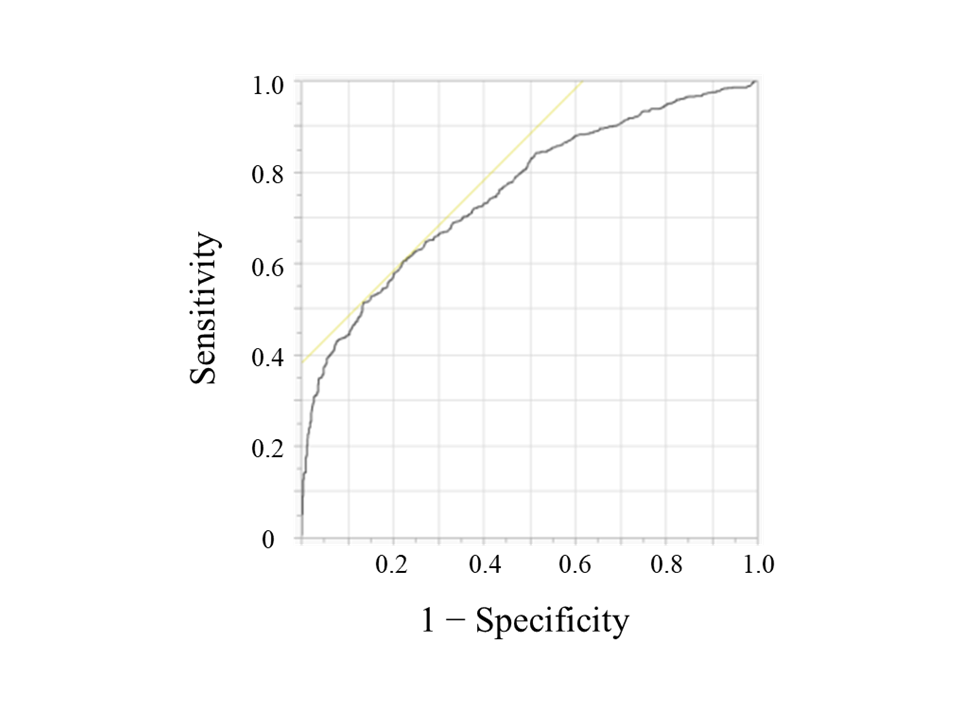

Supplement: Supplementary file 2 — Supplementary Material 2. Figure S1. Receiver operating characteristic curve of propensity score model in Analysis 1. The c-statistic (AUC) was 0.755 [file 12903_2026_8422_MOESM2_ESM.tif]
